# Promoting Diverse Youth’s Career Development through Informal Science Learning: The Role of Inclusivity and Belonging

**DOI:** 10.1007/s10964-022-01694-2

**Published:** 2022-11-07

**Authors:** Mengya Zhao, Channing J. Mathews, Kelly Lynn Mulvey, Adam Hartstone-Rose, Luke McGuire, Adam J. Hoffman, Mark Winterbottom, Angelina Joy, Fidelia Law, Frances Balkwill, Karen P. Burns, Laurence Butler, Marc Drews, Grace Fields, Hannah Smith, Adam Rutland

**Affiliations:** 1 University of Exeter, Exeter, UK; 2 North Carolina State University, Raleigh, NC USA; 3 Cornell University, Ithaca, NY USA; 4 University of Cambridge, Cambridge, UK; 5 Centre of the Cell, Queen Mary University of London, London, UK; 6 Virginia Aquarium & Marine Science Center, Virginia Beach, VA, USA; 7 Thinktank Science Museum, Birmingham, UK; 8 EdVenture, Columbia, SC, USA; 9 Riverbanks Zoo & Garden, Columbia, SC USA; 10 The Florence Nightingale Museum, London, UK

**Keywords:** Inclusivity, Inclusion, Social identity, Belonging, Career development, Informal science

## Abstract

Little research has examined the associations between perceived inclusivity within informal science learning sites, youth program belonging and perceptions of program career preparation. This study explored relations between these factors at three timepoints (T1 = start of program, T2 = 3 months and T3 = 12 months after start). Participants were a diverse sample of 209 adolescents participating in STEM youth programs within informal science learning sites situated in the United States and United Kingdom (70% females: *M*_age_ = 15.27, *SD*_age_ = 1.60), with 53.1% British and 64.1% non-White. Path analysis revealed that only perceptions of inclusivity for own social identity group (i.e., gender, ethnicity) at T1 were associated with T2 STEM youth program belonging. There was a significant indirect effect of T1 perceptions of inclusivity for one’s own social identity groups on T3 perceptions of program career preparation via T2 program belonging. This study highlights that, over time, perceptions of inclusivity around youth’s own social identity groups (i.e., gender and ethnicity/culture) are related to a sense of youth program belonging, which in turn is later associated with perceptions of program career preparation.

## Introduction

Globally, there is a substantial shortage of people entering the Science, Technology, Engineering and Math (STEM) workforce, with the number of skilled STEM graduates not meeting the demand for STEM workers (Peterson et al., 2015). This shortage has its roots in patterns of STEM disengagement during mid-to-late adolescence (approximately 14–16 years old), particularly for girls and those from ethnic minoritized or lower-income groups (Metcalf, 2010). An important obstacle to people from underrepresented groups entering STEM career fields is a lack of inclusivity within many formal science education contexts (Simon et al., 2017). Given the limitations of formal science education, informal science learning sites (e.g., science museums, centers, and zoos) may be a positive and engaging alternative to formal schooling contexts, which could foster STEM career engagement and interests among adolescents (Stocklmayer et al., 2010). Research suggests that youth joining out-of-school or informal science activities tend to show an interest in STEM-related careers in university (Dabney et al., 2012). Informal science learning sites may be a key space for adolescents to feel included in STEM (Hoffman et al., 2021). Research has not made it clear how inclusivity in informal contexts relates to the perceptions of career preparation in youth. This study aims to examine the role of inclusivity and belonging in STEM youth programs at informal science learning sites in order to understand whether these factors are associated with perceptions that these programs help adolescents prepare for their future careers.

### STEM Youth Programs and Youth Career Preparation

STEM youth programs at informal science learning sites involve adolescents enabling the STEM learning of visitors to the sites and gaining support from the sites in pursuing their STEM interests. Recent research supports the benefits for youth participating in STEM youth programs at informal science learning sites, such as increasing youth’s interest and self-efficacy in STEM (Hoffman et al., 2021). Research drawing from the Vocational Anticipatory Socialization model has identified various STEM-related message sources (e.g., school, media, peers, and family) that play a role in young people’s career development (Myers et al., 2011). The Vocational Anticipatory Socialization model has not examined the impact of STEM youth programs on young people’s career preparation. Career preparation is an important developmental task for adolescents (Marciniak et al. 2022), and participation in programs that expose youth to diverse potential career paths is key to career preparation. During this key developmental period, adolescents build the skills and motivation to engage in career preparation (Koivisto et al., 2011).

STEM youth programs within informal science learning sites could play a role in shaping adolescents’ career intentions and prepare them for their future careers. For example, STEM summer programs organized by high schools or universities in the United States do enhance high school students’ STEM career aspirations (Kitchen et al., 2018). It was found that students’ joining such programs involving the real-life relevance of STEM were more likely to report STEM career aspirations compared to either those joining programs that did not focus on the real STEM issues or those not joining any programs. This research suggests that STEM youth programs can foster STEM career development among adolescents, but less is known about the factors that promote such development among diverse youth within informal science learning sites.

### Theoretical Framework

This research utilizes and extends *Social Cognitive Theory* (Bandura, 1992) and *Social Cognitive Career Theory* (Lent et al., 1994). Social Cognitive Theory emphasizes the importance of the interaction between the social environment and individual characteristics (e.g., age, gender, ethnicity) in the process of learning, while recognizing that development among youth is intrinsically related to the social context. Social Cognitive Career Theory (Lent et al., 1994), which builds on Social Cognitive Theory, suggest that the social environment, including its inclusivity and attachment to it, could be related to one’s career development. An important element of the social context is whether informal science learning sites are inclusive and welcoming for youth from under-represented social groups (e.g., girls and youth from ethnic minoritized backgrounds).

### The Role of Inclusivity

Informal science learning sites are concerned about diversity and equity (Kinsley 2016), including gender inclusion (Achiam & Holmegaard, 2017), though informal science learning sites are not always welcoming to youth from all social groups (Dawson, 2014). Inclusivity is often conceived as the promotion of an environment in which underrepresented groups of youth (e.g., females, and people from historically marginalized ethnic/cultural groups) are made to feel welcome (Ainscow & César, 2006). The perceptions of site inclusivity within informal science learning contexts center on youth perceptions of whether the social environment is structured to be welcoming to different social groups. Research has not examined how an inclusive social environment relates to career preparation in youth, but it has investigated whether a critical element of the social environment within schools is associated with career development among adolescents. This research showed that perceptions of the classroom climate among 7^th^ grade students (e.g., teacher expectations, promotion of cooperation and teacher social support) had an indirect effect on later career aspirations in math among 12^th^ grade adolescents (Wang, 2012).

### The Role of Belonging

Belonging to a STEM youth program was defined as a feeling of personal attachment to the youth program within an informal science learning site (Mendoza-Denton et al., 2002). There has been increased attention to belonging within STEM education research and how belonging contributes to STEM interest and academic outcomes, especially in people from historically underrepresented groups (Rainey et al., 2018). A recent study highlights how belonging is related to career interests among both females and males (Xu and Lastrapes, 2021). This study found that among university students a sense of belonging was associated with career interests in male participants and belonging had an indirect effect on female participants’ career interests via STEM attitudes.

Belonging has been associated with positive psychological and social outcomes. For example, STEM youth program belonging was positively associated with STEM interest and self-efficacy amongst youth in the UK and US (Hoffman et al., 2021). A sense of belonging was positively linked with the intention to pursue a math career in female college students (Good et al., 2012), increased computing interest in high school girls (Master et al., 2016), greater STEM class engagement and STEM activism orientation in high school students (Mulvey et al., 2022), higher STEM academic motivations in female graduate students (Smith et al., 2013) and higher career expectations in high school students (Wong et al., 2019). A meta-analytic review has confirmed the positive impact of school belonging on students’ academic achievement, academic engagement, and self-efficacy (Korpershoek et al., 2020). Research does suggest that belonging is related to many positive youth developments, including within the field of STEM, though research has not examined the role of youth program belonging within informal science learning sites in youth career development.

### The Association between Inclusivity and Belonging

Recent research suggests that feeling included is related to a psychological sense of belonging within universities among young people (O’Brien et al., 2020), and within high schools among adolescents (Mulvey et al., 2022). Perceptions of inclusivity could engender a sense of belonging amongst youth at informal science learning sites in two potentially overlapping ways. First, inclusivity could function at the broad and general social-environmental level (i.e., microsystem that directly impacts individuals; Bronfenbrenner, 1992), creating a sense that all people (including those from one’s own and other social identity groups) are welcome at the site, which in turn could promote a positive sense of belonging (Mulvey et al., 2022). For instance, prior research with ethnically diverse adolescents in the United States documented that perceptions of general inclusivity within STEM classes were associated with a sense of belonging in STEM classes, regardless of one’s gender or ethnicity (Mulvey et al., 2022).

Second, perceptions of inclusivity within informal science learning sites may operate at a more specific social identity level (i.e., a feeling that people *like you* are welcome) rather than a more generic sense that ‘all’ are welcome. This proposition is supported by Social Identity Theory (Tajfel and Turner, 1986), which highlights how feelings of shared social identity for your particular identity group are influential to the psychological outcomes in the lives of individuals. This theory would anticipate that youth from underrepresented groups need to feel that their social identity groups are specifically welcome at informal science learning sites, and they may need to see a connection between their own social identity (e.g., gender, ethnicity/culture) and their informal science learning site. Social Identity Theory would anticipate that a sense of personal belonging to the STEM youth program would be more likely if there were a perception of compatibility between the youth’s social identity and an inclusive informal science learning site.

From an individual’s perspective, inclusivity for their own social identity groups may play a more important role compared to inclusivity for other social identity groups. The importance of inclusivity for *your group* may be especially important for youth from historically underrepresented groups, who have often experienced marginalization at informal science learning sites (Dawson, 2014). Inclusivity could be conceptualized in relation to other social identity groups, with the focus on how welcoming a site is for youth from other gender or cultural/ethnic groups than your own. Theoretically, this notion of inclusivity is unlikely to be associated with youth program belonging since it is doubtful that youth will attend as much to how inclusive an environment is for people with whom you don’t share a social identity.

Within this study inclusivity is considered in various ways: general inclusivity (i.e., that the site is welcoming to all gender and cultural/ethnic groups), and specific inclusivity. Specific inclusivity can be divided into two dimensions: inclusivity for youth’s own social identity groups (i.e., individuals perceive that a site is welcoming to those from their own gender or cultural/ethnic groups) and inclusivity for other social identity groups (i.e., individuals perceive that a site is welcoming to those from the different gender or cultural/ethnic groups from them).

## Current Study

Little research has examined the associations between perceived inclusivity within informal science learning sites, youth program belonging and perceptions of program career preparation. This study addresses two research questions. First, drawing on Social Cognitive Career Theory (Lent et al., 1994), the study examines whether inclusivity within informal contexts relates to the perceptions of program career preparation among diverse youth via youth program belonging. Second, it investigates if this pathway is evident when the conceptualization of inclusivity is considered in two ways: general inclusivity as well as specific inclusivity, particularly a sense of inclusivity around one’s own social identity groups. The current research used a survey design to investigate, over time, the factors related to the perceptions of program career preparation in a diverse youth sample (e.g., females and ethnically marginalized youth) who participated in STEM youth programs at informal science learning sites. The study aims to investigate specific hypotheses within two separate models (i.e., one model includes general inclusivity as a broad concept and the other model includes both inclusivity for own social identity groups and inclusivity for other social identity groups). It is anticipated that perceptions of general inclusivity and inclusivity for own social identity groups, but not for the inclusivity for other social identity groups, will be positively associated with perceptions of program career preparation (Hypothesis 1). Second, it is expected that only perceptions of general inclusivity and inclusivity for own social identity groups will be positively associated with program belonging (Hypothesis 2). Third, program belonging is expected to be positively associated with perceptions of program career preparation (Hypothesis 3). Last, it is anticipated that perceptions of site inclusivity will have an indirect impact on perceptions of program career preparation via belonging and that this indirect effect will hold for general inclusivity as well as the inclusivity for one’s own social identity groups (Hypothesis 4).

## Methods

### Participants

The broader study sample included 471 adolescents and young adults from the US and the UK who participated in STEM youth programs and took part in a longitudinal study of adolescent learning and STEM engagement in informal science learning settings. The STEM youth programs in this study were long-term and well-established programs that aim to involve youth in many STEM events or career-related activities. The current study focuses on the perceptions of inclusivity, belonging and career preparation, which take time to form within a youth program. This is why only participants who had meaningful involvement in and exposure to a STEM youth program (i.e., they remained engaged for at least three months and answered the survey) were included in the study.

Only 44.3% of participants (*n* = 209) completed the follow-up survey 3 months after the program started (T2). Participants were aged from 10 to 20 years old at T1 assessment (*M*_*age*_ = 15.27, *SD*_*age*_ = 1.60); 8 participants did not report their age. The sample (69.9% girls and 30.1% boys) was racially and ethnically diverse, with 111 participants (53.1%) from the UK (4.8% of the total sample is White British; 25.8% South Asian British; 7.2% Black British; 5.3% Dual Heritage) and 98 participants (46.9%) from the US (31.1% of the total sample is White/ European American; 3.3% Asian American; 9.1% Black American; 1.9% Hispanic/Latinx; 1.4% Mixed-Race/Bi-racial). Three (1.4%) participants did not report their race or ethnicity. Females and the majority of the non-White ethnic minoritized groups were considered as underrepresented in STEM in the UK (Codiroli Mcmaster, 2017) and the US (Kricorian et al., 2020).

At the beginning of the program, participants were asked to indicate their future career or occupation. Two undergraduate research assistants coded the information (25% of data were checked for inter-rater reliability, Cohen’s kappa = 0.93). 69.8% of participants demonstrated interests in STEM-related career orientations. Specifically, 59.8% of participants reported *only* STEM-related career orientations, and 10% of participants expressed *both* STEM and non-STEM career orientations. 7.7% of participants reported interests in career orientations which are not related to STEM. 22.5% of participants did not report their thoughts about future career occupations or indicated that they were not sure about future career occupation.

Participants were recruited from six informal science learning sites (three from the US and three from the UK). The UK sites include a biomedical and cell biology science education center (37.3%), a science museum (10.5%), and a medical heritage museum (4.3%). The US sites included an aquarium (23.9%), a zoo (14.8%), and a children’s museum (8.1%). Two participants did not report at which site they participated in the STEM education program. Young people serve as youth educators and are trained to interact with visitors, including welcoming visitors to the sites, engaging them in STEM issues and concepts centered around exhibits or animals and facilitating learning at special events or activities at the sites (e.g., one-off exhibits or ‘pop ups’). All the sites have the mission to be inclusive and aim to recruit diverse youth and engage them in STEM to inspire future generations of STEM workers. In addition to increasing young people’s STEM knowledge and equipping them with key soft skills (e.g., communication skills, problem-solving, interpersonal confidence) they provide career advice and support, with the goal of empowering their pursuit of a STEM career and fueling their motivation for STEM as future area of work (e.g., by providing career workshops and supporting College/University applications). None of the youth programs had any time limit, and participants could participate as long as they wish.

### Procedure

Participants were from an Institutional Review Board-approved longitudinal study conducted by a joint research team from the University of Exeter and North Carolina State University. Parents were provided with information about the study prior to their children starting the program. After receiving consent from the participants’ parents, the research team sent a Qualtrics survey to the participants’ email. For those completing the survey, low-value electronic gift cards were sent out as an expression of gratitude.

The longitudinal study was conducted across different time points. The first time point (T1) was the beginning of the program, and the research teams sent the survey out before program activities commenced, but while participants were completing training or orientation. All participants completed the survey within the first month of their program. The second time point (T2) was around 3 months after the start of the program. The third time point (T3) was twelve months after joining the program. The surveys sent at T1 and T3 were the same, and the survey at T2 was different from T1 and T3. The T2 measures were brief and meant to check in on how they were feeling about their participation in the program but did not include the same items at T1 and T3. Participants were asked to complete a smaller bank of measures at T2, focused explicitly on feelings of belonging. This decision was made to reduce participant burden.

### Measures

#### Inclusivity of the site

Four items assessing inclusivity were adapted from previous research (Mulvey et al., 2022) for this study. The questions asked participants to rate how welcoming the site is for gender groups (boys and girls) and ethnic or cultural groups (*own* ethnic/cultural group and *other* ethnic/cultural groups) on a six-point scale (1 = *Not at all welcoming*; 6 = *Very welcoming*). Two items assessing the perceptions of inclusivity related to ethnic cultural groups were used: “How welcoming or not welcoming is X [the site you participated in STEM education program] for *your* ethnic/cultural group?” and “How welcoming or not welcoming is X [the site you participated in STEM education program] for *other* ethnic/cultural group?”. There are two items assessing the perceptions of inclusivity related to gender: “How welcoming or not welcoming is X [the site you participated in STEM education program] for girls?” and “How welcoming or not welcoming is X [the site you participated in STEM education program] for boys?” The total score of the four items is considered as general inclusivity (*α*_T1_ = 0.87; *α*_T3_ = 0.90).

For the specific inclusivity, the items related to gender were recoded according to participants’ gender. The score of inclusivity for own social identity groups was calculated by the total score of the item assessing inclusivity for own ethnic/cultural group and the item assessing inclusivity for own gender (*α*_T1_ = 0.80; *α*_T3_ = 0.74). The score of inclusivity for other social identity groups was calculated by the total score of the item assessing inclusivity for other gender and the item assessing the inclusivity for other ethnic groups (*α*_T1_ = 0.68; *α*_T3_ = 0.80). Inclusivity was not assessed at T2.

#### Belonging in STEM youth program

Eight items adapted from the institutional belonging scale (Mendoza-Denton et al., 2002) were modified to assess belonging in the STEM education program (*α*_T2_ = 0.94). The eight items measured participants’ belonging within their program and their comfort with and connection to their leader and peers in the program (e.g., “How much do you feel that you fit in within your program at X [the program participants joined]?”) based on a 10-point scale (1 = *Definitely do not fit in*; 10 = *Definitely fit in*). Belonging was assessed at T2.

#### Perceptions of program career preparation

One item (i.e., “How much is X [the program participants join] preparing you for your career?”) was used to ask participants to rate their program in terms of their future career preparation on a 6-point scale (1 = *None at all*; 6 = *A lot*). This item was assessed at T1 and T3, but not T2.

### Data Analysis

#### Missing data analyses

Missing data analysis was conducted in STATA 17. First, a frequency analysis was conducted to confirm the percentage of missingness for each variable (Table S1 in supplementary materials), followed by further descriptive analysis to identify the patterns of missing data (Table S2). The mean percentage of missingness at T1, T2, and T3 is 4.8%, 18.1%, and 33.4%, respectively. As shown in Table S2, only around 53% of participants (*n* = 110) answered all items across three time points. Around 36% of participants had missing data at one-time point and around 6% of participants had missing data at two time points.

To describe the mechanism of missingness, whether the missingness was associated with certain variables was examined. All variables were dummy coded with 1 indicating missing data and 0 indicating no missing data. A chi-square test and t-test were conducted to describe the missingness mechanism. Chi-square tests assessed relationships between the missingness and demographic information (e.g., gender, country, minor/major ethnicity and site). For variables at T2 and T3, t-tests were run to investigate if the missingness depended on variables at T1. Results are presented in the supplementary materials (Table S3 and Table S4). In summary, missingness at T1 was not associated with gender, ethnicity, and site. For T2, although missingness did not depend on the variables at T1, the missingness was associated with country and site. As for T3, similar to T2, missingness was not associated with the variables at T1, but the missingness was associated with some demographic variables (i.e., gender, site, ethnicity, and country).

From the missing data analysis, it can be concluded that the distribution of the data is not normal (Table S1), and the missingness of the data is not missing completely at random (MCAR). The missingness is not ignorable and should be addressed for analyses.

#### Multiple Imputation

MPLUS 8.4 (Muthén & Muthén, 2017) was used to run multiple imputation to address missingness. Multiple imputation was carried out using Bayesian analysis (Muthén & Muthén, 2017) and conducted for variables from T1 to T3 with missing values. Specifically, the demographic variables (e.g., gender, site, country, ethnicity) and all variables from T1 to T3 were used to impute ten databases. The imputed databases were used to calculate sum scores for program belonging, inclusivity for own social groups, inclusivity for other social groups, and general inclusivity. Due to missing data on exogenous predictors of the model which are not estimated (i.e., ethnicity), the sample size was reduced to 206 participants for the final path analysis.

#### Path analysis

A path analysis using robust maximum likelihood (MLR) estimation was conducted to examine the hypotheses using MPLUS 8.4 (Muthén and Muthén, 2017). Specifically, the model focused on general inclusivity was computed first, then the model with specific inclusivity was computed (i.e., inclusivity for own social identity groups and inclusivity for other social identity groups). Control variables (i.e., country, ethnicity, gender) were added in the two models to control the impact of the demographic variables on the paths from inclusivity (T1) to perceptions of program career preparation (T3) via program belonging (T2). When controlling for ethnicity, each participant was coded into one of two ethnic categories: participants identifying as White and participants identifying as non-White. In all models, country, gender, and ethnicity were not significantly related to any construct in the model. Mediation analysis was used to explore the indirect effects of inclusivity on perceptions of program career preparation via program belonging.

The model fits for CFA and SEM were evaluated based on a joint consideration of the value of chi-square/degree of freedom (*χ*^*2*^*/df*, ≤5), the values of root mean square error of approximation (RMSEA, ≤0.08), standardised root mean square residual (SRMR, ≤0.06), comparative fit index (CFI, ≥0.90) and Tucker-Lewis index (TLI, ≥0.90) following standard recommendations (Hu & Bentler, 1999; Marsh et al., 2005). Models were tested using the imputed datasets. When conducting path analyses using imputed data, the *p* values are not provided in the MPlus output for model fits.

If the path models demonstrated good model fit, the hypotheses would be evaluated in the following ways. Hypothesis 1 would be supported if there was a significant direct effect of T1 inclusivity (general inclusivity or own social identity groups’ inclusivity) on T3 perceived career preparation. Hypothesis 2 would be supported if there was a significant coefficient from T1 inclusivity (general inclusivity or own social identity groups’ inclusivity) to T2 program belonging. Hypothesis 3 would be supported if there was a significant path from T2 belonging to T3 perceived career preparation. Hypothesis 4 would be supported if there is a significant indirect effect of T1 inclusivity on T3 perceived career preparation via T2 belonging.

## Results

### Model Results for General Inclusivity

The model fit of the path model was not acceptable (computed using the mean of 10 imputed databases): *χ*^*2*^(9) = 24.03, CFI = 0.82, TLI = 0.64, RMSEA = 0.09, SRMR = 0.06. Because the fit was not acceptable, the results were not interpreted further.

### Model Results for Specific Inclusivity

**Fig. 1:**
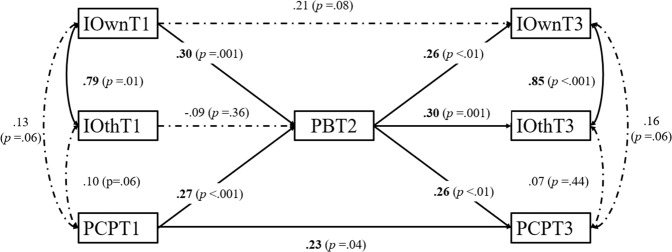
Path analysis results (*n* = 206). Note Values are standardized. IOwn=inclusivity for own social identity groups, IOth = inclusivity for other social identity groups, PB program belonging, PCP perceptions of career preparation; Inclusivity for other social identity groups at T1 and at T3 were not significantly associated with each other. Because the control variables were not significantly associated with any variables, these variables were not included in the Fig 1.

The model fit of the path model for specific inclusivity (Fig. 1) was acceptable: *χ*^*2*^(15) = 32.98, CFI = 0.95, TLI = 0.90, RMSEA = 0.08, SRMR = 0.06.

The correlation matrix can be found in Table 1. We found that ethnicity was correlated with perceptions of inclusivity at T1. Specifically, being White was correlated with higher inclusivity for one’s own social identity groups and inclusivity for other social identity groups. Gender was correlated with perceptions of program career preparation at both T1 and T3. Specifically, identifying as a girl was correlated with lower perceptions of program career preparation.

**Table 1:** Correlation for the model with specific inclusivity (*n* = 209)

	2	3	4	5	6	7	County	Ethnicity	Gender
1 IOwn T1	0.79^***^	0.20^**^	0.11	0.25^***^	0.12	0.06	−0.02	−0.21^**^	−0.03
2 IOth T1		0.12	0.05	0.17^*^	0.10	0.03	−0.04	−0.16^*^	−0.06
3 IOwn T3			0.86^***^	0.27^**^	0.05	0.22^*^	0.10	−0.15	−0.12
4 IOth T3				0.27^**^	−0.01	0.13	0.03	−0.10	−0.08
5 PB T2					0.31^***^	0.33^***^	−0.11	0.03	−0.10
6 PCP T1						0.31^**^	−0.01	0.13	−0.14^*^
7 PCP T3							−0.05	0.06	−0.16^*^

(1) *IOwn* inclusivity for own social identity groups, *IOth* inclusivity for other social identity groups, *PB* program belonging, *FCP* perceptions career preparation, *T1* timepoint 1, *T2* timepoint 2, *T3* timepoint 3

(2) Dummy coding. Country, 0 = US and 1 = UK; Ethnicity, 0 = White, 1 = Non-White; Gender, 0 = Boys, 1 = Girls

(3) *p < 0.05; **p < 0.01; ***p < 0.001

The results can be seen in Fig. 1. The direct effect of T1 inclusivity for own social identity groups on T3 perceptions of program career preparation was not significant, which does not support Hypothesis 1. It was found that participants who perceived a high level of inclusivity for their own social identity groups at T1 were more likely to report stronger program belonging at T2, supporting Hypothesis 2. Similarly, perceiving that the program prepared one more for one’s career at T1 was associated with stronger program belonging at T2. Program belonging at T2 was positively associated with participants’ perceptions of inclusivity for their own social groups and program career preparation at T3, which supported Hypothesis 3. Perceptions of program career preparation at T1 and T3 were positively associated, but the association between inclusivity for one’s own social identity groups at T1 and T3 was not significant. Inclusivity for other social identity groups at T1 was not associated with program belonging at T2 or inclusivity for other social identity groups at T3. Increased program belonging at T2 was positively associated with inclusivity for other social identity groups at T3. Importantly, as predicted, mediation analysis revealed a significant indirect effect of program belonging at T2 on perceptions of program career preparation at T3 through inclusivity for one’s own social identity groups at T1, *β* = 0.08, SE = 0.04, *p* = 0.04, supporting Hypothesis 4. There was a significant indirect effect from perceptions of program career preparation at T1 on perceptions of program career preparation at T3 through program belonging, *β* = 0.07, SE = 0.03, *p* = 0.04.

## Discussion

Despite the importance of social environment on youth career development highlighted by Social Cognitive Career Theory (Lent et al., 1994), little research has focused on the role of inclusivity and belonging in youth’s career preparation within informal science learning contexts. This study examined whether perceptions of inclusivity within informal contexts relate to the perceptions of career preparation via youth program belonging among a diverse sample of adolescents participating in youth programs. It was found that over time only perceptions of inclusivity around youth’s own social identity groups (i.e., gender and ethnicity/culture) were related to a sense of youth program belonging, which in turn was later associated with perceptions of program career preparation. These findings suggest that the social environment plays an important role in the future career preparation of the diverse, and typically underrepresented, youth within our sample. Key to a positive social environment is a perception that the informal science site is inclusive towards youth from the youth’s own social identity groups (i.e., gender, ethnicity/culture) and that the youth program in the site is associated with a sense of belonging among the youth.

The results partially supported the hypotheses. Among the diverse sample of youth from the US and the UK, perceptions of site inclusivity for one’s own social identity groups (i.e., gender and ethnicity/culture) when they began their informal program (T1) were positively associated with a sense of STEM youth program belonging (T2), and this belonging was related to participants’ perceptions of how well the program prepared them for their future career (T3). Findings suggest that only inclusivity for one’s own social identity groups at the beginning of the program was related to participants’ perceptions of program career preparation after one year via belonging, but this mediation pathway did not hold for the inclusivity for other social identity groups. The model for general inclusivity (i.e., perceptions that the site was inclusive for all people, including people like, and not like, me) was a poor fit for the data, indicating that specific types of inclusivity may be more central than general inclusivity at informal science learning sites.

### The Role of Inclusivity

The finding of a positive association between perceptions of site inclusivity and program belonging supports Social Cognitive Theory (Bandura, 1992), since it suggests an inclusive social context within informal science learning sites is associated with the emergence of an individual’s sense of belonging to youth programs. This result extends recent research findings within formal school learning, which documented relations between inclusion and belonging (Mulvey et al. 2022), to the informal science learning context.

Two conceptualizations of inclusivity were explored. First, there was a general inclusivity conceptualization in line with previous theory (Bronfenbrenner, 1992), which contends that perceptions of inclusion for all are key to positive psychological outcomes (i.e., inclusivity is an “umbrella” under which all can flourish). Second, perceptions of inclusivity towards different social identity groups were examined, including inclusivity for one’s own social identity groups (e.g., the context makes my group, those who share the same gender and ethnicity, feel welcome) and inclusivity for other social identity groups (e.g., the context makes other groups, those who do not share my gender and ethnicity, feel welcome). Though Mulvey et al. (2022) documented that general feelings of inclusivity in STEM classes were associated with feelings of belonging in STEM classes, the findings in the current study did not support the association between general inclusivity and belonging in informal settings.

In this study, perceptions of inclusivity for one’s own social identity groups were related to belonging and perceived career preparation (Tajfel & Turner, 1986). There may be several reasons why specific, not general, inclusivity mattered in the context of the present study. First, previous research has looked at formal school contexts, which often promote a strong, well-established, and shared class or school identity that students typically adopt. A generic sense of inclusivity might be seen as applicable to all social groups. This is not the case in informal science learning sites. Compared to schools, at informal science learning sites, learning opportunities are more autonomous. Young people are not required to go to informal science learning sites, and they have more options to choose what they would like to learn in the informal learning context. Second, youth spend less time and have less exposure in informal science learning settings compared to the formal learning context. This means the informal science learning environment may not foster the same type of common group (e.g., school) identity that formal settings encourage, and perceptions of inclusivity that are specific to the youth’s social identities and the social identity groups to which they belong may be more important.

These findings provide a novel insight into how perceptions of inclusivity are related to belonging to a STEM youth program and indicate that feeling that people like you are included at a site may be especially important for promoting feelings of belonging to STEM youth programs at these sites. Conceptually, it is important to explore inclusivity for one’s own group and for other groups, as these may operate differently. In fact, the results supported this distinction, demonstrating the indict effect of inclusivity for own social identity groups to program belonging, but not for other social identity groups. Curiously, there was a high correlation between inclusivity for own social identity groups and for other social identity groups in the current study. All the sites have explicit missions around inclusivity, which may explain this high correlation. Despite this high correlation, inclusivity for own social identity groups in particular appears to be especially important for youth.

Inclusivity was assessed over a one-year period. By T3, participants may have a more fine-tuned (and potentially different) perception of site inclusivity than when they were first beginning a program at the site, as they will have a lot more observations on which to base their perceptions by T3. This may explain why inclusivity was not related at T1 and T3. Future research should examine the psychometric properties of site inclusivity, for example, testing the factor structure of inclusivity. In the current study, an existing measure of inclusivity (Mulvey et al., 2022) was used, and it included only two items for own social identity groups and for other social identity groups, which limits the ability to test a factor model for inclusivity. The development of a more comprehensive measure of inclusivity would allow for factor analysis and a better understanding of what types of inclusivity are most important to different groups and in different contexts (e.g., formal science learning or informal science learning).

### The Role of Belonging

The findings of this study support the important role of belonging within an informal science learning context (Hoffman et al., 2021) and, for the first time, in relation to career development (Good et al., 2012). This study provides evidence that belonging is associated with the perceptions of program career preparation at informal science learning sites, and the findings demonstrate the indirect effect of belonging from inclusivity for one’s own social identity groups to perceptions of program career preparation. This study supports Social Cognitive Career Theory (Lent et al., 1994), which highlights how career development is related to the social environment (i.e., inclusivity) via individual characteristics, such as, in the case of this study, youth program belonging. Social Cognitive Career Theory stresses the role of the social environment in career development via one’s motivation or expectations (e.g., self-efficacy). The findings confirm this but extend Social Cognitive Career theory (Lent et al., 1994) by considering the role of belonging as a critical psychological factor in career development.

The results demonstrated that T1 perceptions of program career preparation (i.e., baseline) were associated with T2 belonging (i.e., after three months). This finding should be interpreted in the context of a STEM youth program that aims to equip youth with the necessary knowledge and skills for education and career development. If participants found the program helpful in career development, they could be more likely to have a higher level of program belonging. This suggests that the association between belonging and career development may be bi-directional. This should be tested in future studies using a more rigorous cross-lagged panel design with additional time points.

### Implications

The findings further extend the formal science education literature by showing the association between inclusivity and perceptions of career preparation within informal science learning contexts (Mulvey et al., 2022). In addition, the study documents the association between belonging and career preparation. Although previous research has highlighted the central role of belonging in STEM career interests (Xu & Lastrapes, 2021) and career development (Good et al., 2012), no study has explicitly shown the relationship between inclusivity, belonging and career development. For example, despite the increasing attention on belonging in the literature (Master and Meltzoff 2020), the literature only documents the impact of belonging on youth’s STEM learning and does not consider the effect on youth career development. The findings suggest that in addition to being concerned with STEM knowledge and career development, STEM youth programs should focus on fostering participants’ sense of belonging to the site, which can be done via inclusive practices, such as building and providing inclusive activities that under-represented youth can connect with and feel that they are fully welcomed (Abrica et al., 2022).

## Limitations

Several limitations need to be noted when interpreting the findings. The sample in the current study included more female participants than male participants and more participants from ethnic minoritized backgrounds than majority backgrounds. While this is a strength of the study, given that these groups are often underrepresented in STEM, future research should continue to explore if these findings are generalizable to other groups. The participants chose to participate in these programs and likely had relatively high levels of pre-existing interests in STEM. While this indicates that the findings may not be generalizable to other populations of young people, this is a novel element of the study and the sample studied is an important one to understand.

Participants were recruited from two countries. Participants from both countries were analyzed together, as adolescents’ psychological processes in both countries should be similar. The sample size is too small to justify a multi-group analysis by country, gender, or ethnic group. Instead, gender and ethnic groups were controlled for in the path models. While the correlations indicated that non-White participants felt the sites were less inclusive to their ethnic group and other ethnic groups and that girls felt less prepared for their careers by the programs, findings from the full model indicated that there were no differences when gender and ethnicity were added to the model as controls. Future work might continue to explore race/ethnicity and gender differences in young people’s perceptions of inclusivity and their sense that STEM programs shape their career preparation.

There was a limitation around the measure of the perceptions of program career preparation in the current study. Perceptions of program career preparation were assessed using a single item. The item did not specify if feelings of career preparation were specifically related to STEM careers, although 69.8% of participants reported STEM-related career interests. Career preparation is a very complex concept, and future studies may wish to use potential career-related questionnaires to assess career preparation (Marciniak et al., 2021).

All measures in this study were self-report, and the variables reported by young people indicate perceptions rather than a more general picture of what happens at informal science learning sites. Future studies could use other assessments of inclusivity, such as exploring inclusive practices and policies in informal science learning contexts. As is common in longitudinal research, there was a good deal of missing data in this study. It should be noted that participants included in the study were those who were still in the program and responded to the survey after three months because research suggests only long-term interventions impact career development (Pfarrwaller et al., 2015). This should be considered when interpreting the findings.

The measures are not repeated measures across time (i.e., inclusivity and career preparation in STEM youth program were measured at T1 and T3, but program belonging was only measured at T2). Cross-lagged panel analysis could be conducted to provide longitudinal evidence of the association between belonging and career development. Future studies should use repeated measures to further test this association. Additionally, future research should explore additional factors that may play a role in these associations. Given the design, this study is not a longitudinal study. It is critical to examine the change in the variables over time to establish longitudinal evidence, which while not the focus of the present work, can be an important future direction.

## Conclusion

Research has not examined some potentially important environmental factors in career preparation during the critical developmental period of adolescence. These factors include youth perceptions of inclusivity within an informal science learning environment and the psychological sense of belonging to a youth program within such an environment. This is despite the fact that Social Cognitive Career Theory (Lent et al., 1994) highlights the importance of the social environment in an individuals’ career development. The findings of this study suggest that a sense of belonging is a key psychological factor that is positively associated with perceptions of program career preparation, and perceptions of inclusivity for youth’s own social identity groups are related to higher belonging in a STEM youth program. This study shows that the social environment is associated with the future career preparation of diverse and underrepresented youth. Important components of a positive social environment for these youth include possessing an awareness that informal science sites are inclusive towards youth from the youth’s own social identity groups (i.e., gender, ethnic/cultural) and having a sense of belonging toward the youth program.

## Supplementary information

Supplementary Tables
