# Supplementary material for: Promoting Diverse Youth’s Career Development through Informal Science Learning: The Role of Inclusivity and Belonging
Source: J Youth Adolesc. 2022 Nov 7;52(2):331–43. doi: 10.1007/s10964-022-01694-2 (PMC9842558; doi:10.1007/s10964-022-01694-2)
Supplement: Supplementary file 1 — Supplementary Tables [file 10964_2022_1694_MOESM1_ESM.docx]

**Table S1.** Descriptive analysis of missing data (*n*=209)

| Variable | N of missingness | Mean | SD | Min | Max |
| --- | --- | --- | --- | --- | --- |
| T1 |  |  |  |  |  |
| IOS_1 | 10 | 5.68 | .54 | 2 | 6 |
| IOS_2 | 10 | 5.67 | .49 | 4 | 6 |
| IOS_3 | 10 | 5.55 | .62 | 2 | 6 |
| IOS_4 | 11 | 5.60 | .54 | 4 | 6 |
| IOS_5 | 10 | 5.68 | .53 | 2 | 6 |
| IOS_6 | 10 | 5.66 | .51 | 4 | 6 |
| PCP | 10 | 4.84 | 1.04 | 1 | 6 |
| T2 |  |  |  |  |  |
| PB_1 | 24 | 8.01 | 1.76 | 1 | 10 |
| PB_2 | 24 | 7.89 | 1.96 | 1 | 10 |
| PB_3 | 23 | 8.38 | 1.82 | 2 | 10 |
| PB_4 | 24 | 8.19 | 1.89 | 2 | 10 |
| PB_5 | 23 | 8.27 | 1.82 | 1 | 10 |
| PB_6 | 25 | 7.91 | 1.92 | 1 | 10 |
| PB_7 | 23 | 8.64 | 1.72 | 1 | 10 |
| PB_8 | 23 | 8.49 | 1.73 | 1 | 10 |
| T3 |  |  |  |  |  |
| IOS_1 | 69 | 5.71 | .49 | 4 | 6 |
| IOS_2 | 70 | 5.69 | .54 | 3 | 6 |
| IOS_3 | 69 | 5.59 | .61 | 3 | 6 |
| IOS_4 | 70 | 5.62 | .52 | 4 | 6 |
| IOS_5 | 69 | 5.72 | .47 | 4 | 6 |
| IOS_6 | 70 | 5.68 | .55 | 3 | 6 |
| PCP | 71 | 4.53 | 1.19 | 1 | 6 |

Note. IOS=inclusivity of site; PB=program belonging; PCP=perceptions of program career preparation; IOS_5 &IOS_6 were recorded variables based on the inclusivity for own gender or other gender.

**Table S2 Missingness patterns** (*n*=209)

| Percent | 1 | 2 | 3 | 4 | 5 | 6 | 7 | 8 | 9 | 10 | 11 | 12 | 13 | 14 | 15 | 16 | 17 | 18 | 19 | 20 | 21 | 22 |
| --- | --- | --- | --- | --- | --- | --- | --- | --- | --- | --- | --- | --- | --- | --- | --- | --- | --- | --- | --- | --- | --- | --- |
| 53% | 1 | 1 | 1 | 1 | 1 | 1 | 1 | 1 | 1 | 1 | 1 | 1 | 1 | 1 | 1 | 1 | 1 | 1 | 1 | 1 | 1 | 1 |
| 27% | 1 | 1 | 1 | 1 | 1 | 1 | 1 | 1 | 1 | 1 | 1 | 1 | 1 | 1 | 1 | 0 | 0 | 0 | 0 | 0 | 0 | 0 |
| 6% | 1 | 1 | 1 | 1 | 1 | 1 | 1 | 0 | 0 | 0 | 0 | 0 | 0 | 0 | 0 | 1 | 1 | 1 | 1 | 1 | 1 | 1 |
| 5% | 1 | 1 | 1 | 1 | 1 | 1 | 1 | 0 | 0 | 0 | 0 | 0 | 0 | 0 | 0 | 0 | 0 | 0 | 0 | 0 | 0 | 0 |
| 3% | 0 | 0 | 0 | 0 | 0 | 0 | 0 | 1 | 1 | 1 | 1 | 1 | 1 | 1 | 1 | 1 | 1 | 1 | 1 | 1 | 1 | 1 |
| <1% | 0 | 0 | 0 | 0 | 0 | 0 | 0 | 1 | 1 | 1 | 1 | 1 | 1 | 1 | 1 | 0 | 0 | 0 | 0 | 0 | 0 | 0 |
| <1% | 1 | 1 | 1 | 1 | 1 | 1 | 1 | 1 | 1 | 1 | 1 | 1 | 1 | 1 | 1 | 1 | 1 | 1 | 1 | 1 | 1 | 0 |
| <1% | 0 | 0 | 0 | 0 | 0 | 0 | 0 | 0 | 0 | 0 | 0 | 0 | 0 | 0 | 0 | 1 | 1 | 1 | 1 | 1 | 1 | 1 |
| <1% | 1 | 1 | 1 | 1 | 1 | 1 | 0 | 1 | 1 | 1 | 1 | 1 | 1 | 1 | 1 | 1 | 1 | 1 | 1 | 1 | 1 | 1 |
| <1% | 1 | 1 | 1 | 1 | 1 | 1 | 1 | 1 | 1 | 1 | 1 | 0 | 1 | 1 | 1 | 1 | 1 | 1 | 1 | 1 | 1 | 1 |
| <1% | 1 | 1 | 1 | 1 | 1 | 1 | 1 | 1 | 1 | 1 | 1 | 1 | 0 | 1 | 1 | 1 | 1 | 1 | 1 | 1 | 1 | 1 |
| <1% | 1 | 1 | 1 | 1 | 1 | 1 | 1 | 1 | 1 | 1 | 1 | 1 | 1 | 0 | 1 | 1 | 1 | 1 | 1 | 1 | 1 | 1 |
| <1% | 1 | 1 | 1 | 1 | 1 | 1 | 0 | 1 | 1 | 1 | 1 | 1 | 1 | 1 | 0 | 1 | 1 | 1 | 1 | 1 | 1 | 1 |
| <1% | 1 | 1 | 1 | 1 | 1 | 1 | 1 | 1 | 1 | 1 | 1 | 1 | 1 | 1 | 0 | 1 | 1 | 1 | 1 | 1 | 1 | 1 |
| <1% | 1 | 1 | 1 | 1 | 1 | 1 | 1 | 1 | 1 | 1 | 1 | 1 | 1 | 1 | 1 | 1 | 1 | 1 | 0 | 0 | 1 | 1 |

Note. 1 means complete, 0 means missing. 1= IOS_6 at TP1; 2= IOS_5 at TP1; 3= PCP at TP1; 4=IOS_1 at TP1; 5= IOS_2 at TP1; 6= IOS_3 at TP1; 7= IOS_4 at TP1; 8=PB_3; 9 = PB_5; 10 = PB_7; 11 =PB_8; 12 = PB_1; 13 = PB_2; 14 = PB_4; 15 = PB_6; 16 = IOS_5 at TP2; 17 = IOS_1 at TP2; 18 = IOS_3 at TP2; 19 = IOS_6 at TP2; 20 = IOS_2 at TP2; 21 = IOS_4 at TP2; 22 = PCP at TP2.

IOS=inclusivity of site; PB=program belonging; PCP=perceptions of career preparation; IOS_5 &IOS_6 were recorded variables based on the inclusivity for own gender or other gender.

**Table S3 Chi Square test results** (*n*=209)

|  | Gender  (*df*=1) | | Cohort  (*df*=2) | | Country  (*df*=1) | | Site  (*df*=5) | | Ethnicity  (*df*=1) | |
| --- | --- | --- | --- | --- | --- | --- | --- | --- | --- | --- |
| Variable | *χ^2^* | *p* | *χ^2^* | *p* | *χ^2^* | *p* | *χ^2^* | *p* | *χ^2^* | *p* |
| T1 |  |  |  |  |  |  |  |  |  |  |
| IOS_1 | .51 | .47 | 4.73 | .09 | 1.20 | .27 | 4.36 | .50 | 1.22 | .25 |
| IOS_2 | .51 | .47 | 4.73 | .09 | 1.20 | .27 | 4.36 | .50 | 1.22 | .27 |
| IOS_3 | .51 | .47 | 4.73 | .09 | 1.20 | .27 | 4.36 | .50 | 1.22 | .27 |
| IOS_4 | .79 | .37 | 3.49 | .18 | 1.79 | .18 | 5.57 | .35 | 1.67 | .20 |
| IOS_5 | .51 | .47 | 4.73 | .09 | 1.20 | .27 | 4.36 | .50 | 1.22 | .27 |
| IOS_6 | .51 | .47 | 4.73 | .09 | 1.20 | .27 | 4.36 | .50 | 1.22 | .27 |
| PCP | .51 | .47 | 4.73 | .09 | 1.20 | .27 | 4.36 | .50 | 1.22 | .27 |
| T2 |  |  |  |  |  |  |  |  |  |  |
| PB_1 | 3.17 | .08 | **21.41** | <.001 | **14.46** | <.001 | **19.21** | .002 | 1.46 | .23 |
| PB_2 | 1.71 | .19 | **26.60** | <.001 | **14.46** | <.001 | **18.75** | .002 | 1.46 | .23 |
| PB_3 | 2.18 | .14 | **23.69** | <.001 | **16.66** | <.001 | **21.11** | .001 | 1.97 | .16 |
| PB_4 | 1.71 | .19 | **21.94** | <.001 | **14.46** | <.001 | **18.75** | .002 | 1.46 | .23 |
| PB_5 | 2.18 | .14 | **23.69** | <.001 | **16.66** | <.001 | **21.11** | .001 | 1.97 | .16 |
| PB_6 | 2.59 | .11 | **20.44** | <.001 | **12.50** | <.001 | **16.71** | .005 | 1.04 | .31 |
| PB_7 | 1.04 | .31 | **23.69** | <.001 | **16.66** | <.001 | **21.11** | .001 | 1.97 | .16 |
| PB_8 | 2.18 | .14 | **23.69** | <.001 | **16.66** | <.001 | **21.11** | .001 | 1.97 | .16 |
| T3 |  |  |  |  |  |  |  |  |  |  |
| IOS_1 | **6.91** | .009 | 3.15 | .21 | **21.27** | <.001 | **25.89** | <.001 | **4.45** | .035 |
| IOS_2 | **6.37** | .012 | 4.10 | .13 | **19.87** | <.001 | **24.40** | <.001 | **3.97** | .046 |
| IOS_3 | **6.91** | .009 | 3.15 | .21 | **21.27** | <.001 | **25.89** | <.001 | **4.45** | .035 |
| IOS_4 | **8.08** | .004 | 2.90 | .23 | **19.87** | <.001 | **24.40** | <.001 | **3.97** | .046 |
| IOS_5 | **6.91** | .009 | 3.15 | .21 | **21.27** | <.001 | **25.89** | <.001 | **4.45** | .035 |
| IOS_6 | **6.37** | .012 | 4.10 | .13 | **19.87** | <.001 | **24.40** | <.001 | **3.97** | .046 |
| PCP | **9.33** | .002 | 2.60 | .27 | **23.91** | <.001 | **29.57** | <.001 | **4.75** | .029 |

Note: Bold means significant χ^2^.

IOS=inclusivity of site; PB=program belonging; PCP=perceptions of career preparation; IOS_5 &IOS_6 were recorded variables based on the inclusivity for own gender or other gender

**Table S4 t-test result** (*n*=209)

| T1 | IOS_1 | | IOS_2 | | IOS_3 | | IOS_4 | | IOS_5 | | IOS_6 | | PCP | |
| --- | --- | --- | --- | --- | --- | --- | --- | --- | --- | --- | --- | --- | --- | --- |
| Variable | *t* | *p* | *t* | *p* | *t* | *p* | *t* | *p* | *t* | *p* | *t* | *p* | *t* | *p* |
| T2 |  |  |  |  |  |  |  |  |  |  |  |  |  |  |
| PB_1 | -.62 | .54 | -.78 | .44 | -.11 | .92 | -.45 | .65 | -.58 | .56 | -.81 | .42 | 1.38 | .17 |
| PB_2 | -.62 | .54 | -.78 | .44 | -.10 | .92 | -.45 | .65 | -.58 | .56 | -.81 | .42 | 1.81 | .07 |
| PB_3 | -.50 | .62 | -.64 | .52 | .06 | .95 | -.28 | .78 | -.46 | .65 | -.68 | .50 | 1.66 | .10 |
| PB_4 | -.62 | .54 | -.78 | .44 | -.10 | .92 | -.45 | .65 | -.58 | .56 | -.81 | .42 | 1.59 | .11 |
| PB_5 | -.50 | .62 | -.64 | .52 | .06 | .95 | -.28 | .78 | -.46 | .65 | -.68 | .50 | 1.66 | .10 |
| PB_6 | .06 | .95 | -.04 | .97 | .44 | .66 | .18 | .86 | .12 | .91 | -.09 | .93 | 1.75 | .08 |
| PB_7 | -.50 | .62 | -.64 | .52 | .06 | .95 | -.28 | .78 | -.46 | .65 | -.68 | .50 | 1.66 | .10 |
| PB_8 | -.50 | .62 | -.64 | .52 | .06 | .95 | -.28 | .78 | -.46 | .65 | -.68 | .50 | 1.66 | .10 |
| T3 |  |  |  |  |  |  |  |  |  |  |  |  |  |  |
| IOS_1 | .31 | .76 | .43 | .66 | -1.68 | .09 | -.67 | .50 | .13 | .90 | .62 | .53 | 1.24 | .22 |
| IOS_2 | .22 | .83 | .33 | .74 | -1.78 | .08 | -.79 | .43 | .04 | .97 | .52 | .60 | 1.22 | .23 |
| IOS_3 | .31 | .76 | .43 | .66 | -1.68 | .09 | -.67 | .50 | .13 | .90 | .62 | .53 | 1.24 | .22 |
| IOS_4 | .49 | .62 | .64 | .53 | -1.54 | .13 | -.51 | .61 | .32 | .75 | .82 | .42 | 1.22 | .23 |
| IOS_5 | .31 | .76 | .43 | .66 | -1.68 | .09 | -.67 | .50 | .13 | .90 | .62 | .53 | 1.24 | .22 |
| IOS_6 | .22 | .83 | .33 | .74 | -1.78 | .08 | -.79 | .43 | .04 | .97 | .52 | .60 | 1.22 | .23 |
| PCP | .13 | .90 | .23 | .82 | -1.64 | .10 | -.63 | .53 | -.05 | .96 | .42 | .68 | 1.33 | .18 |

Note. Two tail test; Difference = mean of not missing – mean of missing; As for degree of freedom, only IOS_4’s df=196, others’ df=197;

IOS=inclusivity of site; PB=program belonging; PCP=perceptions of career preparation; IOS_5 &IOS_6 were recorded variables based on the inclusivity for own gender or other gender.
